# Factors associated with loss to follow-up after occupational HIV exposure in Cape Town, South Africa: a retrospective cohort study

**DOI:** 10.1186/s12981-017-0149-8

**Published:** 2017-04-21

**Authors:** Nectarios Sophocles Papavarnavas, Kathryn Manning, Fahd Conrad, Milah Govender, Gary Maartens

**Affiliations:** 1 Department of Medicine, Old Main Building, J-Floor, Groote Schuur Hospital, University of Cape Town, Observatory, Cape Town, 7925 South Africa; 2 Trauma and Emergency Unit, Floor E11A, Management Suite, Groote Schuur Hospital, Main Road, Observatory, Cape Town, 7925 South Africa; 3 Quality and Safety Unit, Groote Schuur Hospital, Main Road, Observatory, Cape Town, 7925 South Africa; 4 Division of Clinical Pharmacology, Department of Medicine, Groote Schuur Hospital, University of Cape Town, Cape Town, 7925 South Africa

**Keywords:** Loss to follow-up, Post exposure prophylaxis, Health care workers, HIV

## Abstract

**Background:**

There is limited data on factors associated with loss to follow-up (LTFU) of health care workers (HCWs) following occupational exposure to HIV, and most studies were conducted in an era when poorly tolerated antiretrovirals like zidovudine were used.

**Methods:**

A retrospective cohort study was conducted of HCWs attending a referral hospital’s Occupational Health Clinic in Cape Town, South Africa for post-exposure prophylaxis (PEP) during a period when tenofovir was available. Our primary outcome was LTFU at the 3-month visit. We selected seven variables a priori for our logistic regression model and ensured there were at least 10 outcome events per variable to minimize bias.

**Results:**

Two hundred and ninety-three folders were evaluated for descriptive analysis. LTFU worsened with successive visits: 36% at 6 weeks, 60% at 3 months, and 72% at 6 months. In multivariate analysis at the 3-month visit LTFU was associated with age (adjusted odds ratio (aOR), 0.6 per 10-year increase [95% CI, 0.5–0.9]), HCW category of doctor (aOR 2.7 [95% CI, 1.3–5.5]), and time from exposure to receiving PEP of more than 24 h (aOR 5.9 [95% CI, 1.3–26.9]).

**Conclusion:**

We identified factors associated with LTFU of HCWs after occupational HIV exposure, which could be used to target interventions to improve follow-up.

## Background

The World Health Organization (WHO) estimates 3 million occupational human immunodeficiency virus (HIV) percutaneous exposures occur among 35 million healthcare workers (HCWs) annually, with 90% of exposures occurring in resource limited settings [1]. HCWs exposed to potentially infectious material from a source patient with HIV infection, or unknown HIV status, are offered post-exposure prophylaxis (PEP) if this is appropriate, and attend several follow-up visits for PEP toxicity monitoring and exclusion of HIV infection. Although HIV seroconversion following occupational exposure is uncommon, early diagnosis is critical as treatment of early HIV infection reduces the risk of HIV transmission and has direct benefits for the individual by reducing morbidity and mortality [2, 3].

Attendance of HCWs to follow-up visits after occupational exposure to HIV has been highly variable, ranging from 0 to 98.9% in observational studies [4–13]. A literature search yielded three studies that evaluated factors associated with attendance to follow-up [6, 12, 13]. Two studies found that the type of HCW category did not influence attendance to follow-up, [12, 13] but one study found that HCW category did influence attendance to follow-up [6]. One study identified being exposed to an HIV seropositive source increased attendance to follow-up, [13] but another found this had no effect [6]. One study [13] reported that type of exposure and time to reporting did not influence attendance to follow-up; and women had better attendance to follow-up than men [13]. Sample size calculations were not reported in any of these three studies. Furthermore, many of the PEP regimens used in these studies included poorly tolerated antiretroviral drugs. A recent systematic review reported that tenofovir disoproxil fumarate (TDF) based PEP was better tolerated with higher completion rates than zidovudine based PEP, which used to be the standard of care [14]. The WHO now recommends the use of TDF as part of the backbone for PEP regimens [15].

We aimed to evaluate factors associated with loss to follow-up (LTFU) following occupational exposure to HIV in a referral hospital in Cape Town, South Africa, where the HIV prevalence is 12.7% [16]. We conducted our study in a period when we switched to TDF based PEP, and ensured we had sufficient power to determine variables associated with LTFU.

## Methods

### Study setting

Data was collected from the Occupational Health Clinic (OHC) of Groote Schuur Hospital, a referral hospital in Cape Town, South Africa. HCWs who have significant occupational HIV exposure are started on PEP if they present within 72 h of the exposure. During the study period the South African national guidelines for PEP recommended the use of TDF and emtricitabine for exposures presenting within 72 h. The policy of adding a 3rd antiretroviral (usually a boosted protease inhibitor) was changed during the study period: initially this was added only for high risk exposures, but subsequently this was added for all exposures. HCWs are counselled about the risks of HIV, the need to document HIV testing for possible compensation, and potential adverse drug reactions to PEP. HIV status of the HCW was determined at baseline using 4th generation Roche COBAS HIV-1/2 Combo automated test with a confirmatory Siemens Integral 4th generation ELISA. PEP was discontinued in HCWs who tested HIV seropositive at baseline. HIV testing was repeated at week 6 (when HCWs were informed that HIV tests may be false negative), and months 3 and 6 after the exposure. Confidentiality of HCWs is protected by keeping all files in the OHC and not in the general records department; only OHC clinic staff are able to access the files.

### Study design

We conducted a retrospective cohort review to identify the factors associated with LTFU in HCWs following occupational HIV exposure. The OHC maintains an electronic database of all visits related to HCW occupational HIV exposures. We collected data from the database and additional data from folders between January 2013 and September 2015, with 29 additional folders obtained in 2012.

### Outcomes

The primary outcome was the proportion of HCWs LTFU at the 3-month visit, which is the key follow-up date to determine if HIV seroconversion has occurred, in keeping with the WHO guidelines [15]. Secondary outcomes were the proportion of HCWs LTFU at the 6-week and 6-month visits.

### Data collection

Data collection at the OHC is collated from an “Occupational Health Clinic Percutaneous Inoculation Report” form. The data are collected each time the HCW presents for follow-up or when contacted by telephone. This form is completed by the occupational health worker on duty, who is either the nurse or doctor. Once 6 months have lapsed since the last visit, the data collected are recorded in an electronic database using Microsoft Excel and HCW folders are then archived in the clinic.

### Study population

#### Inclusion criteria

HCWs were categorized into three groups: ‘Doctors’, ‘Students’ and ‘Allied Health Professionals’ (e.g. nursing, physiotherapists, occupational therapists, administrative clerks, pharmacists, and emergency medical services). HCWs were included if they were exposed to potentially infectious material from patients who are HIV-infected or HIV status unknown and attended the OHC. The following materials were deemed to be potentially infectious: pleural, pericardial, peritoneal, cerebrospinal, synovial fluid, amniotic fluid, and blood [17].

#### Exclusion criteria

Exclusion criteria were: HCW tested HIV seropositive at baseline; exposed to a HIV seronegative source; those who requested follow-up at a private doctor or who went back to their own training institutions, such as elective students; multiple exposures within the 6-month follow-up period; exposures deemed to be from non-infectious material [17].

#### Sample size estimation

We selected seven variables a priori for inclusion into our model based on our review of the literature: age at exposure, sex, HCW category, type of exposure, source patient HIV status, dual or triple antiretroviral therapy (ART), and time from exposure to time of receiving PEP. Assuming 25% LTFU at 3 months, [12] we required a sample size of 280 to ensure a minimum of 10 outcome events per variable, which are needed to improve precision and minimize bias in logistic regression models [18].

### Statistical analysis

All statistical analysis was performed using Stata (Version 13.1; Stata Corp, College Station, Texas, USA). Descriptive statistics were used to characterize the total sample, and results were expressed as median (interquartile range) for non-normally distributed continuous variables, and frequencies and percentages for categorical variables.

We used separate multivariable logistic regression models for each time point to identify factors associated with LTFU at the 3-month, 6-week, and 6-month follow-up visits. The full model approach was utilized using a priori selected variables in order to ensure decreased risk of selection bias and overfitting [19]. This approach allows multiple epidemiological variables to be assessed independently while controlling effects of other variables [20]. Univariate analysis was used to estimate crude odds ratios (ORs) and 95% confidence intervals (95% CIs), while multivariable logistic regression provided adjusted estimates for odds of LTFU at each time point. Odds ratios were presented with 95% CIs and a level of *P* < 0.05 was considered statically significant.

## Results

**Fig. 1:**
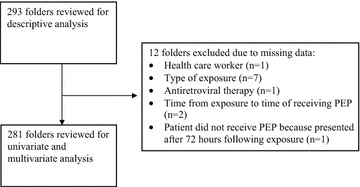
Flow chart illustrating data set of health care workers chosen for analysis

Two hundred and sixty-four folders were obtained between January 2013 and September 2015, with an additional 29 folders collected according to alphabetical order in 2012. There was incomplete data on 12 HCWs who were included in the descriptive analysis but excluded from the univariate and multivariate analysis as shown in Fig. 1.

The characteristics of the 293 patients from our cohort are shown in Table 1. The dual nucleotide/nucleoside reverse transcriptase inhibitors used in PEP were TDF (97%), zidovudine (2%), and stavudine (1%); all of which were combined with either lamivudine or emtricitabine. The third agent used in 210 HCWs who were given triple ART was lopinavir/ritonavir (82%), atazanavir/ritonavir (13%), raltegravir (4%), and efavirenz (1%).

**Table 1:** Baseline characteristics and follow-up of 293 health care workers with occupational exposure

Variable	Sample (n)
Age, median (IQR)	28 (24–35)
Sex	
Women	197 (67%)
Men	96 (33%)
Health care worker^a^	
Allied health professional	85 (29%)
Doctor	100 (34%)
Student	107 (37%)
Type of exposure^b^	
Hollow-bore	137 (48%)
Mucocutaneous	86 (30%)
Solid sharp	63 (22%)
Source patient HIV status	
Positive	246 (84%)
Unknown	47 (16%)
Antiretroviral^c,f^	
Dual	81 (28%)
Triple	210 (72%)
Time from exposure to receiving PEP^d^ (h)	
<24	268 (92%)
24–48	17 (6%)
48–72	1 (0.3%)
>72	4 (1.4%)
Loss to follow-up^e^	
6 weeks	100 (36%)
3 months	169 (60%)
6 months	203 (72%)

Number with missing data: ^a^ n = 1, ^b^ n = 7, ^c^ n = 1, ^d^ n = 2, ^e^ n = 12

^f^ One health care worker did not receive PEP because presented too late

LTFU at the various visits were: 36% at 6 weeks, 60% at 3 months, and 72% at 6 months (Table 1). The univariate and multivariate analysis of variables associated with LTFU at the 3-month visit are shown in Table 2. In the multivariate analysis, significant risk factors associated with LTFU were: younger age, HCW category of doctor, and time from exposure to receiving PEP of more than 24 h. The multivariate analysis of variables associated with LTFU at the 6-week and 6-month follow-up visits are shown in Table 3. Variables associated with LTFU at the 6-week visit were: male sex and HCW category of doctor. Variables associated with LTFU at the 6-month visit were similar to the 3-month visit: younger age, HCW category of doctor, and time from exposure to receiving PEP of more than 24 h.

**Table 2:** Variables associated with loss to follow-up at 3 months

Variables	Unadjusted OR (95% CI)	P value	Adjusted OR (95% CI)	P value
Age (per 10-year increase)	0.7 (0.5–0.9)	0.003	0.6 (0.5–0.9)	0.011
Sex				
Women ^a^				
Men	1.4 (0.8–2.4)	0.190	1.4 (0.8–2.5)	0.262
Health care worker				
Allied health professional^a^				
Doctor	2.9 (1.6–5.4)	0.001	2.7 (1.3–5.5)	0.006
Student	2.0 (1.1–3.6)	0.022	1.2 (0.6–2.6)	0.584
Type of exposure				
Hollow-bore^a^				
Mucocutaneous	1.6 (0.9–2.9)	0.095	1.1 (0.6–2.2)	0.707
Solid sharp	1.3 (0.7–2.4)	0.377	1.0 (0.5–1.9)	0.948
Source patient HIV status				
Positive	0.9 (0.4–1.8)	0.742	0.5 (0.2–1.1)	0.074
Unknown^a^				
Antiretroviral				
Dual	1.4 (0.8–2.3)	0.250	1.5 (0.8–2.8)	0.228
Triple^a^				
Time from exposure to receiving PEP (h)				
<24^a^				
>24	3.0 (1.0–9.2)	0.052	5.9 (1.3–26.9)	0.023

^a^ Reference category

**Table 3:** Variables associated with loss to follow-up at 6 weeks and 6 months

Variables	6-week		6-month	
	Adjusted OR	P value	Adjusted OR	P value
Age (per 10-year increase)	1.0 (0.7–1.3)	0.764	0.6 (0.5–0.9)	0.010
Sex				
Women^a^				
Men	1.8 (1.1–3.2)	0.027	1.8 (0.9–3.4)	0.082
Health care worker				
Allied health professional^a^				
Doctor	2.1 (1.1–4.4)	0.034	2.1 (1.0–4.5)	0.049
Student	1.1 (0.5–2.5)	0.750	1.3 (0.6–2.9)	0.532
Type of exposure				
Hollow-bore^a^				
Mucocutaneous	1.0 (0.5–1.9)	0.988	1.2 (0.6–2.5)	0.659
Solid sharp	1.1 (0.5–2.1)	0.857	1.1 (0.5–2.4)	0.728
Source patient HIV status				
Positive	1.0 (0.5–2.2)	0.952	0.7 (0.3–1.7)	0.489
Unknown^a^				
Antiretroviral				
Dual	1.2 (0.6–2.3)	0.544	0.7 (0.4–1.4)	0.333
Triple^a^				
Time from exposure to receiving PEP (h)				
<24^a^				
>24	1.3 (0.5–3.4)	0.552	7.8 (1.0–61)	0.049

^a^ Reference category

**Fig. 2:**
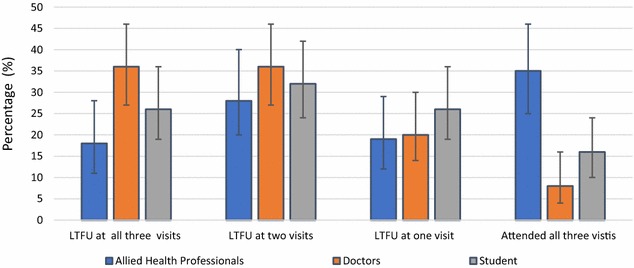
Percentage loss to follow-up (LTFU) by number of visits of various health care worker categories (visits were scheduled at 6 weeks, 3 months, and 6 months).* Error bars* are shown

LTFU by category of HCW at the various visits is shown in Fig. 2, with doctors having the highest proportion LTFU.

## Discussion

We showed that LFTU of HCWs after occupational HIV exposure was high and increased with successive visits. Younger age, the HCW category doctor, and time from exposure to receiving PEP of more than 24 h were associated with LTFU at the 3-month visit, which was our primary endpoint. Men were more likely to be LTFU at the 6-week visit than women. These findings could be used to target interventions designed to improve follow-up.

Our finding that LTFU increases with successive visits is consistent with other studies [9, 12, 13]. We found that younger age was a significant risk factor for LTFU, which is in keeping with other studies [21–23]. The higher LTFU in younger HCWs may be related to the greater change and instability they experience in their younger years [24]. Men tended to be more likely to be LTFU in our study, which is similar to the findings of Escurdero et al. [13].

The majority (207 out of 281) of HCWs in our study were doctors and students. Doctors and students are often involved in invasive medical procedures, which places them at risk of being exposed to infectious material [7, 9, 11, 25]. Furthermore, students and doctors with less than a year’s experience, are prone to occupational exposure because of their inexperience [7, 9, 26]. We found that doctors are more likely to be LTFU than other HCW categories. This could be explained by the ease with which doctors can submit their own blood samples for HIV testing instead of attending the OHC. One study, [27] showed a large proportion of HCWs obtained HIV testing outside of the facility where they worked, which they suggested was due to concern surrounding the confidentiality of HIV testing at the facility. Furthermore, doctors may be making their own assessment of the severity of the exposure and may deem it unnecessary to follow-up [28]. In contrast to our findings of increased LTFU in doctors, Gutierrez et al. [6] showed cleaning personnel were more likely to be LTFU. Two other studies found type of HCW category did not influence attendance to follow-up [12, 13].

Longer time from exposure to receiving PEP at the 3-month visit was positively associated with LTFU. This could be explained by HCWs who present after 24 h having a perceived lower benefit from PEP. However, type of exposure and source patient HIV status, which are associated with risk of HIV acquisition, were not associated with LTFU in our cohort.

Escudero et al. [13] also found that type of exposure was not related to attendance to follow-up. Findings from studies that assessed the effect of the source patient’s HIV status on LTFU are contradictory, with one study reporting no effect, [6] while another found positive serological status was associated with improved follow up [13].

There are a number of studies which reported that dual ART regimens are better tolerated than triple regimens [29, 30]. However, many of these studies include ART that are no longer used due to toxicity. Newer studies have shown that completion of PEP is based on the tolerability of ART and not on whether dual or triple therapy are used [31, 32]. This could explain why in our cohort there was no correlation between type of ART used and LTFU.

There were several limitations of our study. First, the retrospective cohort design is inherently prone to bias. However, the data was captured by OHC staff on a standard form and we had very little missing data. Second, although we found that time from exposure to receiving PEP was associated with LTFU, the 95% CIs were wide due to the small sample size of HCWs with delayed presentation. Third, we did not explore associations between years of HCW experience and exposure as we did not have this data. Other researchers have reported an association between years of experience and the incidence of occupational exposures [7, 9, 25]. Finally, Groote Schuur Hospital is a tertiary facility with referrals from other hospitals that fall under the University of Cape Town, so our findings may not be generalizable to other settings such as district hospitals.

We have identified factors associated with LTFU, which could be used to target interventions to decrease LTFU. In one study, [13] contacting HCWs by telephone or mail improved attendance to follow-up from 33 to 54%. Schmid et al. [33] suggested attendance to follow-up could be improved with fewer follow-up visits. The WHO has recently advised the final follow-up visit should be at 3 months rather than 6 months [15]. Furthermore, it has been suggested that the last follow-up visit should be at 6 weeks if laboratory 4th generation HIV ELISA tests are utilized, and 8 weeks if 4th generation rapid HIV tests are utilized [34]. Lastly van der Maaten et al. [9] suggested increasing awareness of the availability of PEP through campaigns.

## Conclusion

We have identified factors associated with LTFU of HCWs after occupational HIV exposure. Future research should identify measures to improve attendance to follow-up, which could be targeted at doctors, younger HCWs, and HCWs with delayed presentation.

## Abbreviations

ART: antiretroviral therapy
CIs: confidence intervals
HCW: health care worker
HIV: human immunodeficiency virus
LTFU: loss to follow-up
OHC: Occupational Health Clinic
ORs: odds ratios
PEP: post exposure prophylaxis
TDF: tenofovir disoproxil fumarate
WHO: World Health Organization
